# Editorial: Neuroinflammation in the interaction between aging and chronic brain injury

**DOI:** 10.3389/fnagi.2023.1283438

**Published:** 2023-09-11

**Authors:** Xintong Ge

**Affiliations:** ^1^Department of Geriatrics, Tianjin Medical University General Hospital, Tianjin, China; ^2^Tianjin Geriatrics Institute, Tianjin, China

**Keywords:** chronic brain injury, aging, neurodegeneration, neuroinflammation, cognitive

Neuroinflammation plays a fundamental role in mediating the onset and progression of aging, especially cellular senescence of the central nervous system. It is also a hallmark of neurodegenerative diseases and chronic brain injury, which could finally lead to cognitive impairment. In all these conditions, the cellular and molecular mechanisms underlying the pathological progress are still expected to be fully elucidated. Herein, two system review and two clinical studies provided new insights into the pathogenesis of brain aging and chronic brain injury, and the potential early-diagnostic and therapeutic strategies for impaired neurological function.

The first systematic review (Lin et al.) highlighted the relationship between diabetes mellitus (DM) and cognitive impairment. Chronic peripheral hyperinsulinemia and insulin resistance are the main features of DM. Meanwhile, hyperglycemia is increasingly thought to be the cause of cognitive impairment in elderly patients with DM. Therefore, the authors systematically analyzed whether glycemic control could improve cognitive function in patients with DM, hyperglycemia, or insulin resistance. The results showed that glycemic control through antidiabetic treatment correlates with the improvement of cognitive function. Positive blood glucose management could attenuate the potential cognitive decline in the patients.

The second meta-analysis studied the association between serum uric acid levels and neurological prognosis in patients with acute ischemic stroke. Uric acid is an abundant and potent endogenous antioxidant that effectively scavenges reactive nitrogen and oxygen radicals. However, it also has prooxidative property in various pathological conditions, and acts as a double-edged sword for humans (Sautin and Johnson, 2008). Conflicting findings of the relationship between serum uric acid and prognosis in acute ischemic stroke have been reported. For the above reasons, this dose-response meta-analysis enrolled 13 cohort studies with 10,485 acute ischemic stroke patients, and aimed to clarify the difference in the risk of poor neurological outcome, hemorrhagic transformation, and post-stroke depression in patients with the highest serum uric acid levels and those with the lowest levels. Overall, serum uric acid levels were not found to associate with the risk of mortality and symptomatic intracerebral hemorrhage. Consequently, further randomized, controlled trials need to be conducted to determine whether maintaining serum uric acid at an appropriately higher level during the acute phase could improve the functional outcome in patients with acute ischemic stroke.

The two retrospective clinical studies both focused on the prognostic indicators for hemorrhagic transformation after intravenous thrombolysis in patients with acute ischemic stroke. Chen et al. suggested that elevated levels of serum bilirubin, including total bilirubin, direct bilirubin, and indirect bilirubin, were independently linearly associated with the risk of hemorrhagic transformation and symptomatic intracranial hemorrhage in patients with acute ischemic stroke undergoing intravenous thrombolysis. Xu et al. found that the ratio of C-reactive protein to Albumin (RCA) was an independent risk factor for both hemorrhagic transformation and poor neurological outcome. From this, future studies are needed to clarify the underlying pathological mechanism in order to identify specific therapeutic targets for controlling serum bilirubin and inflammation related high RCA level.

While the four articles have enriched our understanding of the early-diagnosis for impaired neurological function after chronic brain injury, especially acute ischemic stroke, the exact role of neuroinflammation in the onset and development of brain aging and chronic brain injury still remain to be elucidated. Further research from *in-vitro* to *in-vivo* models are warranted using new techniques, such as single cell sequencing, spatial transcriptomics, and multi-omics analysis, with the hope of exploring potential therapeutic strategies for clinical application.

## Author contributions

XG: Writing—original draft, Writing—review and editing.
